# Tennis Play Intensity Distribution and Relation with Aerobic Fitness in Competitive Players

**DOI:** 10.1371/journal.pone.0131304

**Published:** 2015-06-22

**Authors:** Ernest Baiget, Jaime Fernández-Fernández, Xavier Iglesias, Ferran A. Rodríguez

**Affiliations:** 1 Sport Performance Analysis Research Group, University of Vic, Barcelona, Spain; 2 Sports Research Centre, Miguel Hernandez University, Elche, Spain; 3 INEFC-Barcelona Sports Sciences Research Group, Institut Nacional d’Educació Física de Catalunya, Universitat de Barcelona, Barcelona, Spain; University of Rome, ITALY

## Abstract

The aims of this study were (i) to describe the relative intensity of simulated tennis play based on the cumulative time spent in three metabolic intensity zones, and (ii) to determine the relationships between this play intensity distribution and the aerobic fitness of a group of competitive players. 20 male players of advanced to elite level (ITN) performed an incremental on-court specific endurance tennis test to exhaustion to determine maximal oxygen uptake (VO_2max_) and the first and second ventilatory thresholds (VT_1_, VT_2_). Ventilatory and gas exchange parameters were monitored using a telemetric portable gas analyser (K4 b^2^, Cosmed, Rome, Italy). Two weeks later the participants played a simulated tennis set against an opponent of similar level. Intensity zones (1: low, 2: moderate, and 3: high) were delimited by the individual VO_2_ values corresponding to VT_1_ and VT_2_, and expressed as percentage of maximum VO_2_ and heart rate. When expressed relative to VO_2max_, percentage of playing time in zone 1 (77 ± 25%) was significantly higher (p < 0.001) than in zone 2 (20 ± 21%) and zone 3 (3 ± 5%). Moderate to high positive correlations were found between VT_1_, VT_2_ and VO_2max_, and the percentage of playing time spent in zone 1 (r = 0.68–0.75), as well as low to high inverse correlations between the metabolic variables and the percentage of time spent in zone 2 and 3 (r = -0.49–0.75). Players with better aerobic fitness play at relatively lower intensities. We conclude that players spent more than 75% of the time in their low-intensity zone, with less than 25% of the time spent at moderate to high intensities. Aerobic fitness appears to determine the metabolic intensity that players can sustain throughout the game.

## Introduction

Tennis has evolved from a sport in which skill was the primary prerequisite for successful performance into a sport that also requires complex interaction of several physical components (i.e., strength and agility) and metabolic capacities (i.e., aerobic and anaerobic) [1,2]. Nowadays, several previous studies have reported the external and internal load demands of tennis play [3 **–** 9]. Regarding to the external load, tennis match play is characterized by intermittent whole body efforts, alternating short (2–10 s) bouts of high-intensity exercise and short (10–20 s) recovery bouts interrupted by several resting periods of longer duration (60–90 s) [1,6]. The typical duration of a tennis match is usually 1.5–2 hours but it can last even over 4 hours, and the average rally duration lasts 5–10 s, with a 20 s break, and 60 to 120 s break during the changeovers [1,5,6,10]. Matches comprise about 300–500 high intensity efforts with stroke rates ranging between 2.5–4.7 shots/rally, dependent on gender and surface [1].

From a physiological point of view, during competitive matches, mean heart rate (HR) values ranges between 60–80% of maximum HR (HR_max_), with long and intense rallies eliciting values over 95% of HR_max_ [11], and the oxygen uptake ($\dot{V}$O_2_) values averaged 50–60% of maximal values ($\dot{V}$O_2max_) [1,5]. Average blood lactate concentration ranges from 1.7 to 3.8 mmol·l^-1^, but during long and intense rallies lactate values can go up to 8.6 mmol·l^-1^ [1,5,11]. The rate of perceived exertion (RPE) has been reported as ranging from 5–7 arbitrary units (CR-10) and 10–16 (Borg 20-point) [7,12,13].

Although the successful performance in tennis cannot be defined by a predominating physical attribute, as it involves a complex interaction of physical factors [1,14], it has been suggested that aerobic fitness (i.e., $\dot{V}$O_2max_) is an important component of tennis performance. $\dot{V}$O_2max_ values >50 ml·kg^-1^·min^-1^ are generally considered necessary for competing at a high level [5,6,15,16]. It seems that a good aerobic fitness level enables the player not only to repeatedly generate explosive actions, such as strokes and on-court movements, but also ensures fast recovery between rallies, especially during long matches [16 **–** 18].

As previously described, the common approach for the determination of the intensity during tennis match play has been using maximal values and percentages of $\dot{V}$O_2max_ and HR_max_ [3,4,10,12,19]. However, these relative values may correspond to a wide range of individual exercise intensities [20]. For instance, at the same percentage of $\dot{V}$O_2max_ or HR_max_ some individuals may be above and others below their metabolic (“anaerobic”) threshold—no matter they are determined using ventilatory changes or blood lactate [21]. Therefore, the use of given percentage values of $\dot{V}$O_2max_ or HR_max_ has been questioned when used to determine exercise intensities for training and research purposes [20,22]. To describe the level of physical exertion under competitive conditions, the division of three intensity phases (or zones) according to reference values obtained during physiological testing has been used in different continuous sports [23 **–** 25]. However, information related to the description of these intensity zones in intermittent sports is scarce [26], and no previous study analysed this topic in tennis.

Therefore, the aims of the study were (i) to describe the relative intensity of simulated tennis play based on the cumulative time spent in three intensity zones delimited by ventilatory thresholds (VTs zones) and HR demarcation points (HR zones) identified via an on-court tennis specific incremental test, and (ii) to determine the relationships between this play intensity distribution and the aerobic fitness of a group of high-level competitive players. Our working hypothesis was that the largest part of playing time will be spent below the zone defined by the first ventilatory threshold, and only a small part will be played above the second ventilatory threshold. We also hypothesized that an association will exist between the time spent in VTs and HR intensity zones and the aerobic fitness of tennis players (i.e., $\dot{V}$O_2max_ and ventilatory thresholds), so that players with better aerobic fitness would play at relatively lower intensities.

## Material and Methods

### Subjects

20 male competitive tennis players (mean ± SD; age: 18.0 ± 1.2 years; height: 179.0 ± 8.4 cm; body mass: 71.9 ± 9.5 kg; 75% right handed) with an International Tennis Number (ITN) ranging from 1 (elite) to 3 (advanced) (ITN 1 = 5 players; ITN 2 = 9 players; ITN 3 = 6 players), volunteered to participate in the study. The mean training background of the players was 6.6 ± 2.0 years and the training regimen was 5 d·week^-1^ with a training volume of 23 ± 1.4 h·week^-1^. Players were focusing 3.2 ± 0.3 h·day^-1^ on tennis-specific training (i.e. technical and tactical skills), and 1.4 ± 0.2 h·day^-1^ on aerobic and anaerobic training (i.e. on-court and off-court exercises), and strength training. During competitive periods, the subjects were involved between 2–3 times per month in regular tennis competition (i.e., national tennis circuits and “International Tennis Federation Futures” tournaments). 15% of the participants were left-handed. The study was performed in accordance with current ethical standards [27], and conformed to the recommendations of the Declaration of Helsinki. All subjects voluntarily participated in the study after being informed about the scope and methods of the study, and delivered a written informed consent, with parental permission when needed. Approval for the project was obtained from the Research Ethics Committee of the National Institute of Physical Education, Generalitat de Catalunya.

### Experimental design

In order to delimit the metabolic intensity zones during simulated tennis matches, all participants performed an incremental tennis-specific endurance field test [15], which was recently shown to be reliable and valid for the determination of $\dot{V}$O_2max_ and VT. Two weeks after the field test, the participants played simulated tennis matches (i.e., 20 sets overall). All tests and simulated tennis matches were performed on an outdoor tennis court (i.e., GreenSet surface, GreenSet Worldwide S.L., Barcelona, Spain). During simulated matches, ventilatory gas exchange and HR were continuously recorded using a portable gas analyser and HR monitors. Before any baseline testing, all participants attended two familiarization sessions to introduce the testing procedures and to ensure that any learning effect was minimal for the study measures. To reduce the interference of uncontrolled variables, all the subjects were instructed to maintain their habitual lifestyle and normal dietary intake before and during the study. The subjects were told not to exercise the day before a test and to consume their last (caffeine-free) meal at least 3 h before the scheduled test time.

### Specific endurance tennis test

**Fig 1:**
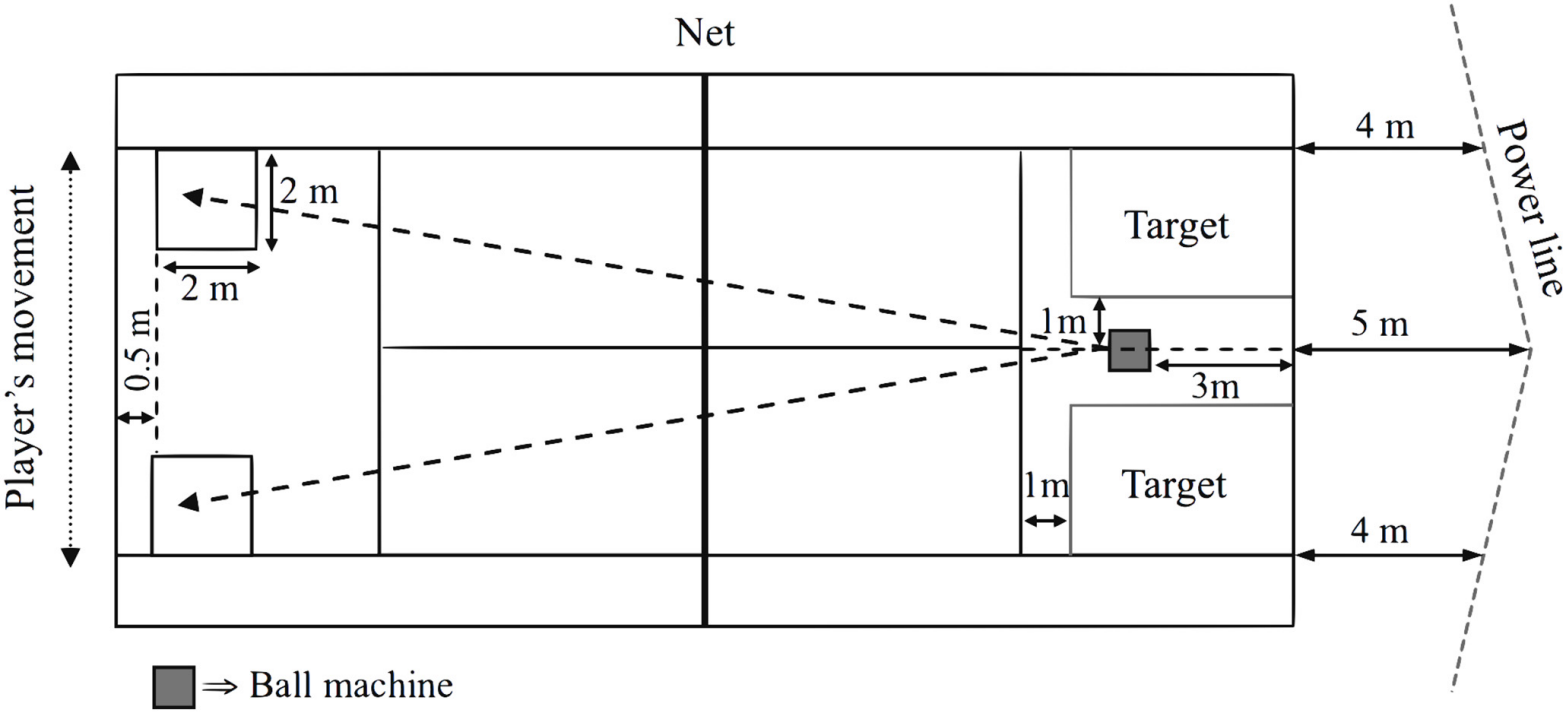
Schematic setting for the specific endurance field test [15].

The test procedure has been described elsewhere [15]. Shortly, participants had to hit balls coming from a ball machine (Pop-Lob Airmatic 104, France), alternating forehand and backhand strokes, cross-court or down the line in a prescribed pattern (i.e., drive, topspin). The landing point for the balls was chosen about 2 m in front of the baseline, alternating balls to the right and the left corners (Fig 1). The test began with a ball frequency of 9 shots·min^-1^, which was increased by 2 shots·min^-1^ every 2 min. The test ended at the player’s request or stopped by the researchers if the player was no longer able to fulfil the test criteria (i.e., the player was no longer able to perform strokes with acceptable stroke technique and precision, determined by the experienced researchers, through subjective observation). In addition to the physiological measurements, an objective evaluation of the technical effectiveness (TE) was carried out. TE was calculated based on the percentage of hits and errors, and two performance criteria were defined: (1) precision: the ball returned by the player had to bounce inside the target (i.e., 3.1 by 4.5 m square located 1 m from the service line and 1 m over the prolongation of the centre service line), and (2) power: once the ball was bouncing inside the target, it had to go over the power line (located between 5 m from the centre of the baseline and 4 m from the side line), before bouncing a second time. A hit was considered successful when both performance criteria were fulfilled at once (precision and power). A minimum of 40 new tennis balls (Babolat Team) was used for each test. The ball machine was manually calibrated before each test and the device’s reliability was assessed by manual timing (mean CV of ball frequency = 3.5 ± 0.9%) and using a radar device (Stalker ATS 4.02, USA) (mean ball velocity = 68.6 ± 1.9 km·h^-1^; CV = 2.7%).

### Physiological measurements

Ventilatory breath-by-breath gas exchange and five-second HR values were continuously recorded, beginning 2 min before the familiarization phase and finishing 5 min after the end of the test (recovery phase). Expired air was analyzed continuously for gas volume (Triple digital-V1 turbine), oxygen concentration (zirconium analyzer), and carbon dioxide concentration (infrared analyzer) using a portable gas analyzer (K4 b^2^, Cosmed, Italy). The portable measurement unit was carried by the player in the same way during all tests. Heart rate monitoring (Polar S610, Kempele, Finland) was used alongside the portable unit of the gas analyzer. Gas and volume calibration of the measurement device was done before each test session. Room air calibration occurred before each test. VTs detection was done by analysing the points of change in slope or breaks in linearity of ventilatory parameters [28]. Two VTs were determined independently by two experienced observers according to the model proposed by Skinner and MacLellan [29]: VT_1_ or first VT, and VT_2_ or second VT (Wasserman’s respiratory compensation point). VT_1_ was determined using the criteria of an increase in the ventilatory equivalent for oxygen ($\dot{V}$_E_ / $\dot{V}$O_2_) with no increase in the ventilatory equivalent for carbon dioxide ($\dot{V}$_E_ / $\dot{V}$CO_2_) and the departure from linearity of $\dot{V}$_E_, whereas VT_2_ corresponded to an increase in both $\dot{V}$_E_ / $\dot{V}$O_2_, and $\dot{V}$_E_ / $\dot{V}$CO_2_. $\dot{V}$O_2max_ was determined by the observation of a “plateau” or levelling off in $\dot{V}$O_2_ or when the increase in two successive periods was less than 150 mL·min^-1^ [28]. HR_max_ was considered as the highest value reached during the final minute of the test.

Three main relative intensity zones were defined using a three-phase model based on ventilatory parameters (VTs zones) [23]: Zone 1 (low intensity; $\dot{V}$O_2_ at or below VT_1_), Zone 2 (moderate intensity; $\dot{V}$O_2_ between VT_1_ and VT_2_), and Zone 3 (high intensity; $\dot{V}$O_2_ at or beyond VT_2_). This triphasic model uses the HR response associated to reproducible metabolic demarcation points (i.e., lactate or ventilatory thresholds), thus allowing to examine the physiological strain during various types of exercise. It has consistently been used in continuous sports [23 **–** 25] and in team sports like soccer [26,30,31]. The HR-based model was previously used in tennis and defines three HR zones [12]: Zone 1 (low intensity; < 70% HR_max_), Zone 2 (moderate intensity; 70–85% HR_max_), and Zone 3 (high intensity; > 85% HR_max_).

### Simulated tennis set

Two weeks after the on-court test players performed a simulated tennis set. Thus, in a time frame of 4 days, all subjects played one set against an opponent of similar level, resulting in 20 sets overall. During play the participants were equipped with a portable gas analyser (K4 b^2^, Cosmed, Italy) and HR monitor (Polar S610, Kempele, Finland). A set of four new balls was used for each set. Sets were played in accordance with the current rules of the International Tennis Federation (ITF) [32] on an acrylic surface (Green set; ITF category 3), at similar time of the day (12:08 ± 02:14 h), with a stable environmental and wind conditions (mean ambient temperature 14.9 ± 4°C, air velocity < 2 m·s^-1^, relative humidity 54.4–61.0%). Before each set, subjects performed a standardised warm-up for five minutes, which included ground strokes (players were asked to play the balls to the centre of the court), volleys plus over head plays (one player on the baseline, the other playing volleys), and services. Both coaches and researchers encouraged the players to exert themselves to their maximum during the games and to simulate the real match play conditions as possible.

### Statistical analyses

The Kolmogorov-Smirnov test was used to ensure a gaussian distribution of the data. Mean values (± SD) were calculated for each of the variables analysed. The differences between the relative intensity calculated by the two triphasic models were tested by paired Student t-tests. Repeated-measures ANOVA was used to compare the percentage of time (% time) spent in each of the three relative intensity zones over tennis matches. The Tukey test was used as a post hoc test. Pearson product—moment correlation coefficients were used to test whether there was a significant relationship between percentage of time in each of the three intensity zones over the tennis sets, and the players’ aerobic fitness parameters (i.e., VT_1_, VT_2_ and $\dot{V}$O_2max_). The level of significance was set at p < 0.05. The magnitude of the differences in mean was shown as effect size and interpreted according to the criteria used by Cohen [33]: <0.2 = trivial, 0.2–0.4 = small, 0.5–0.7 = moderate, >0.7 = large. All statistical analyses were performed using SPSS for Windows 15.0 (SPSS Inc., USA).

## Results

### Specific endurance tennis test

Table 1 shows the values of the physiological and performance parameters corresponding to the intensity at which $\dot{V}$O_2max_ and the ventilatory thresholds (VT_1_ and VT_2_) were attained. For TE, a total of 206 ± 27 hits were made per test, of which 66.5 ± 5.9% were considered to be successful.

**Table 1:** Physiological and performance variables corresponding to $\dot{V}$O_2max_, first ventilatory threshold (VT_1_) and second ventilatory threshold (VT_2_).

	VT_1_	% of maximum	VT_2_	% of maximum	$\dot{V}$O_2max_
Time elapsed (min:s)	06:22 ± 01:07	46 ± 9	10:20 ± 00:55	74 ± 7	13:17 ± 01:38
Stage (#)	2.9 ± 0.6	47 ± 11	4.9 ± 0.5	73 ± 9	6.37 ± 0.80
$\dot{V}$O_2_ (mL·min^-1^)	2695 ± 490	64 ± 4	3575 ± 600	85 ± 2	4199 ± 671
$\dot{V}$O_2_ (mL·kg^-1^·min^-1^)	37.2 ± 4.0	65 ± 5	49.4 ± 9.8	84 ± 7	58.0 ± 4.6
$\dot{V}$CO_2_ (mL·min^-1^)	2444 ± 308	52 ± 7	3566 ± 537	75 ± 5	4838 ± 735
$\dot{V}$_E_ (L·min^-1^)	66 ± 8	50 ± 8	96 ± 10	71 ± 8	136 ± 18
HR (beats·min^-1^)	152 ± 13	79 ± 5	177 ± 9	92 ± 3	189.5 ± 9.6
R	0.90 ± 0.06	80 ± 6	1.00 ± 0.08	88 ± 6	1.14 ± 0.10

Indication of the percentages regarding the maximum values of each variable determined in the specific tennis test (n = 20).

Data are mean ± SD. VT_**1**_, first ventilatory threshold; VT_**2**_, second ventilatory threshold; $\dot{V}$O_**2max**_, maximal oxygen uptake; $\dot{V}$O_**2**_, oxygen uptake; $\dot{V}$CO_**2**_, carbon dioxide production; $\dot{V}$_**E**_, ventilation; HR, heart rate; R, respiratory exchange ratio.

### Simulated tennis sets

**Fig 2:**
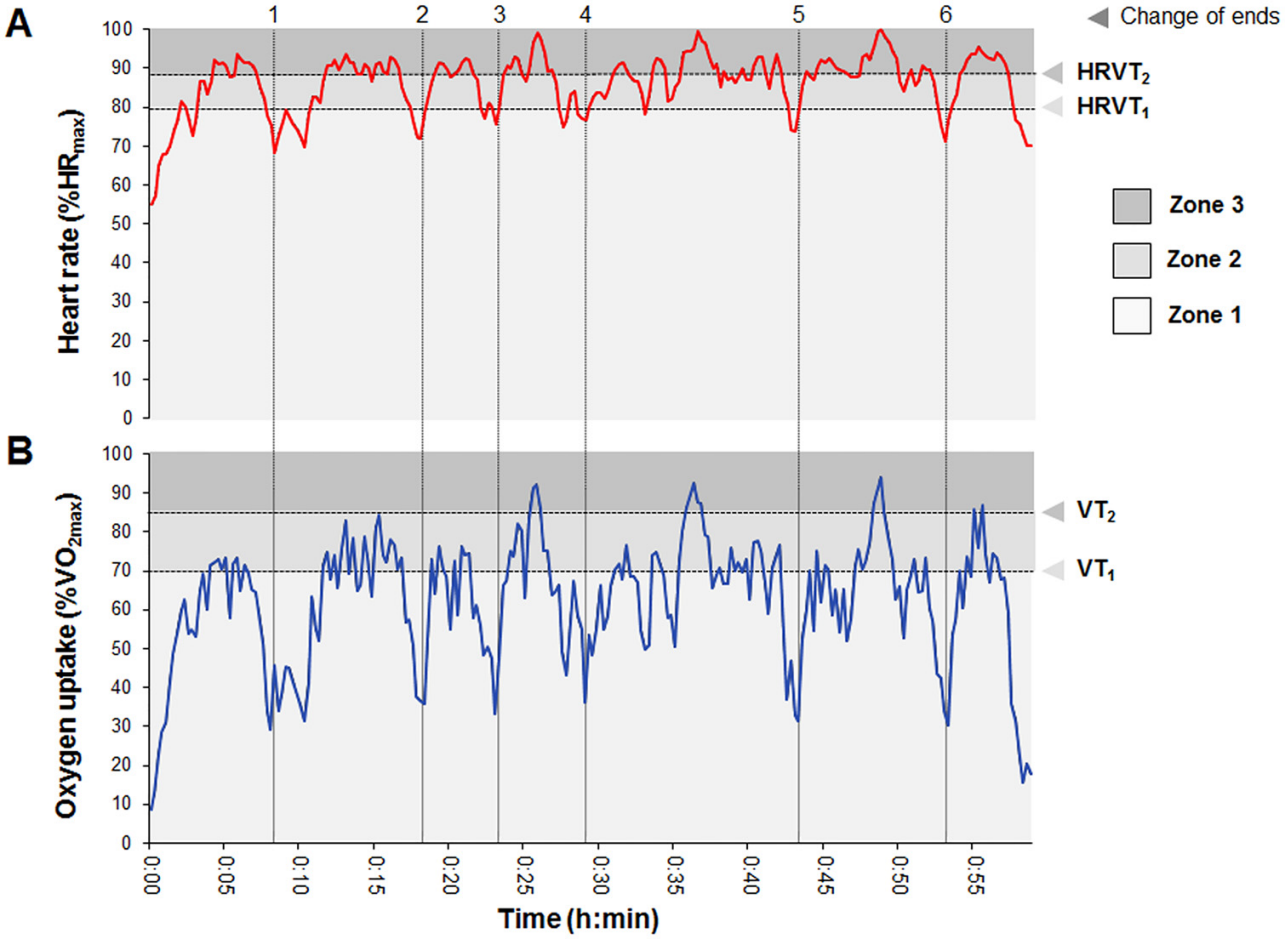
Heart rate (A) and oxygen uptake (B) intensity profiles during one set. Data from a representative subject expressed as percentage of maximum (%HR_max_ and %$\dot{V}$O_2max_, respectively). Three intensity zones are delimited by the first and second ventilatory thresholds, expressed as (A) heart rate (HRVT_1_, HRVT_2_), and (B) $\dot{V}$O_2_ (VT_1_, VT_2_) are illustrated (light to dark grey background). The dashed vertical lines represent the 6 rest periods corresponding to game end changes.

The 20 sets played resulted in a total of 170 games for statistical analyses. The mean duration of the sets was 31:03 ± 11:23 min:s. 56% of the games and 70% of sets played were won by the player who was not carrying the gas analyser. Fig 2 shows an example of $\dot{V}$O_**2**_ and HR kinetics from a representative player during a set with a final score of 7/5.

The average $\dot{V}$O_2_ during the sets ranged from 6.9 ± 2.8 to 47.9 ± 4.7 mL·kg^-1^·min^-1^ and corresponded to 12 ± 5% to 83 ± 11% of $\dot{V}$O_2max_, 14 ± 6% to 97 ± 15% of VT_2_ and 18 ± 8% to 129 ± 20% of VT_1_. The mean HR (Table 2) corresponded to 70 ± 8% of HR_max_, and ranged from 90 ± 19 to 167 ± 18 beats·min^-1^ and corresponded to 47 ± 9% to 86 ± 8% of HR_max_, and to 47 ± 10% to 88 ± 8% HR at $\dot{V}$O_2max_, 51 ± 10% to 94 ± 11% of HR at VT_2_ and 59 ± 12% to 110 ± 16% of HR at VT_1_.

**Table 2:** Physiological values recorded during the simulated sets.

	Mean values	%$\dot{V}$O_2max_	% VT_2_	% VT_1_
$\dot{V}$O_2_ (mL·min^-1^)	2151 ± 363	52 ± 9	61 ± 11	81 ± 15
$\dot{V}$O_2_ (mL·kg^-1^·min^-1^)	29.9 ± 3.7	52 ± 9	61 ± 11	81 ± 15
$\dot{V}$CO_2_ (mL·min^-1^)	1996 ± 313	42 ± 8	57 ± 10	83 ± 15
$\dot{V}$_E_ (L·min^-1^)	54.9 ± 8.6	40 ± 6	57 ± 10	84 ± 15
HR (beats·min^-1^)	138 ± 15	73 ± 7	77 ± 10	90 ± 13
R	0.94 ± 0.03	83 ± 8	95 ± 8	104 ± 7

Relative intensity is shown as a function of submaximal and maximal values in the specific endurance tennis test (n = 20).

Data are mean ± SD. $\dot{V}$O_2_, oxygen uptake; $\dot{V}$O_2max_, maximal oxygen uptake; VT_1_, first ventilatory threshold; VT_2_, second ventilatory threshold; $\dot{V}$CO_2_, carbon dioxide production; $\dot{V}$_E_, ventilation; HR, heart rate; R, respiratory exchange ratio.

**Fig 3:**
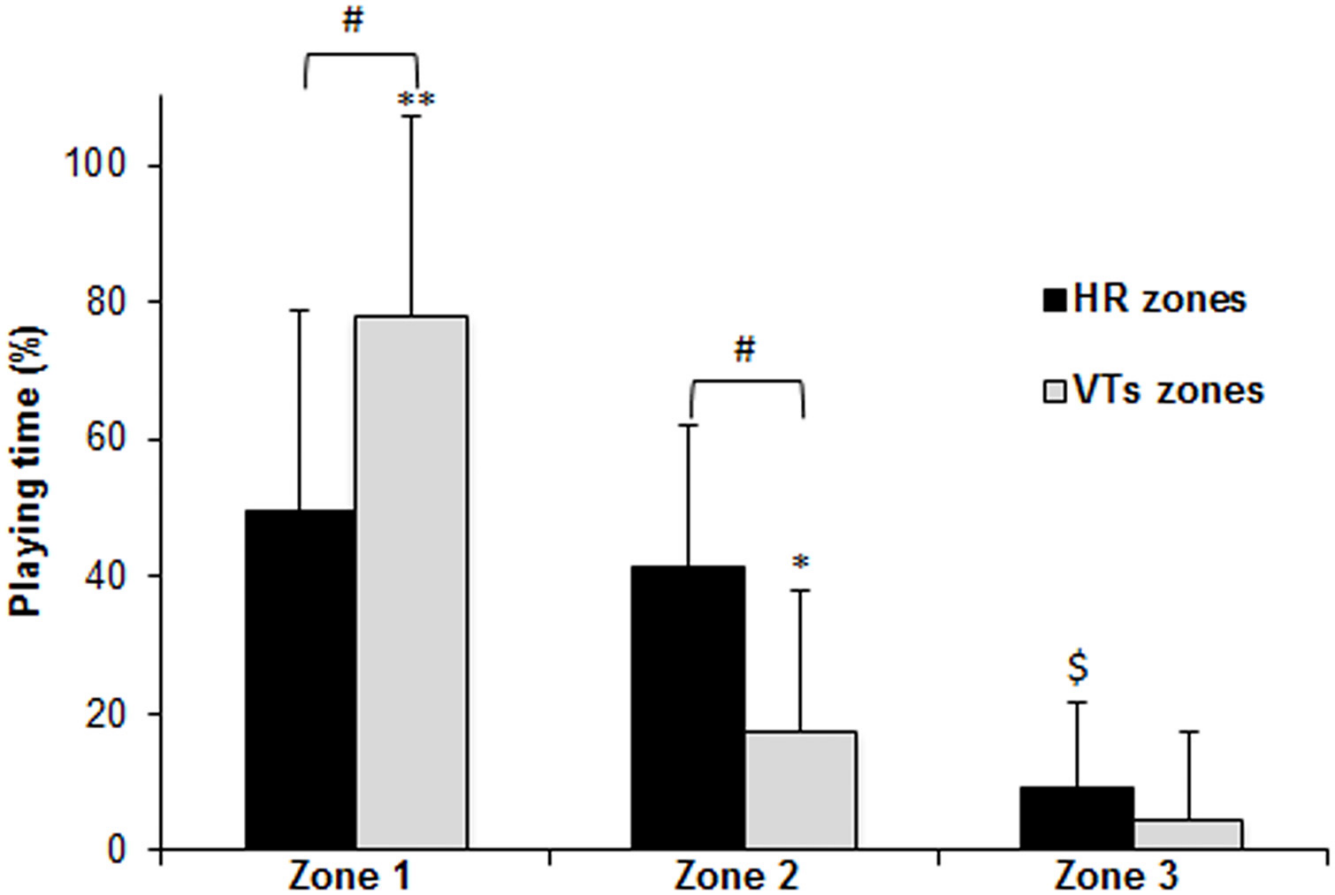
Comparison of playing time (%) spent in the three differentiated intensity zones. Intensity zones defined by the VTs zones method (below VT_1_ (Zone 1), between VT_1_ and VT_2_) (Zone 2), and over VT_2_ (Zone 3)) and defined by the HR zones method (below 70% HR_max_ (Zone 1), between 70 and 85% HR_max_ (Zone 2), and over 85% HR_max_ (Zone 3)). Mean ± SD values and standard deviations. **p < 0.001 for VTs zone 1 vs both VTs zones 2 and 3; *p < 0.05 for VTs zone 2 vs VTs zone 3. $ p < 0.001 for HR zone 3 vs both HR zones 1 and 2. # p < 0.001 for HR vs VTs zones 1 and 2.

Significant differences were found between the intensity zones defined by the two methods (VTs vs. HR zones) for zones 1 and 2 (p < 0.001) (Fig 3), with large effect sizes (0.99 and 1.24, respectively). If we consider the intensity zones defined by the VTs, during most of the playing time, players were at zone 1 (low intensity) (22:14 ± 08:26 min:s), with a shorter time spent at zone 2 (moderate intensity) (07:14 ± 09:25 min:s), and achieving zone 3 (high intensity) only for a very short period of time (01:00 ± 01:55 min:s). Mean % time spent in each of the three intensity zones are shown in Fig 3. Significant differences were found between % time in zone 1 (77.1 ± 24.5%) and % time in zones 2 (20.0 ± 21.2%) and 3 (2.9 ± 4.7%) (p < 0.001) and between % time in zone 2 and % time in zone 3 (p < 0.05), all of them with large effect sizes (2.4, 3.2 and 0.8, respectively). If we consider the intensity zones defined by the HR zones method (Fig 3), no differences were found between the time spent at zone 1 (low intensity) (13:40 ± 07:18) and at zone 2 (moderate intensity) (13:00 ± 08:09) (p > 0.05), and there was a moderate effect size (0.5). Significant differences were found between % time in zone 3 (high-intensity) (04:10 ± 09:25; 9.1 ± 17.0%) and % time in zones 1 (49.6 ± 27.5%) and 2 (41.3 ± 17.7%) (p < 0.001), with large effect sizes (2.3 and 1.9, respectively).

### Relationship between physiological variables, competitive level and intensity zones

Correlations between physiological and technical parameters (VT_**1**_, VT_**2**_, $\dot{V}$O_**2max**_, and TE), competitive level (ITN) and the % time spent at intensity zones during simulated play are presented in Table 3. If we consider the VTs zones method, there was a positive correlation between the physiological variables analysed and the % time spent in zone 1, with significant inverse correlations between these variables and the % time spent in zones 2 and 3. In addition, an inverse relationship (r = -0.65; p = 0.005) was found between players’ $\dot{V}$O_**2max**_ and the %$\dot{V}$O_**2max**_ attained during play. If we consider the HR zones method, no correlations were found between the physiological variables and the % time spent in the three zones. Likewise, no correlations were found between the TE and the % time in any of the zones defined by either method. Regarding to the players’ competitive level, if we consider the VTs zones method, there was a positive correlation between tennis expertise level (ITN) and the % time spent in VT zone 3 (r = 0.55; p = 0.023), no correlations were found between the ITN and the % time spent in VTs zones 1 and 2. If we consider the HR zones method, no correlations were found between the ITN and the % time spent in the three zones. No correlations were found between the ITN and the physiological variables ($\dot{V}$O_**2max**_, VT_**2**_ and VT_**1**_) (r = -0.35, 0.41, 0.21; p > 0.05).

**Table 3:** Pearson’s correlation coefficient (r) between physiological and technical variables determined in the specific tennis test, competitive level and playing time (%) spent in the three differentiated zones.

Variables	Playing time					
	VTs zones method			HR zones method		
	Zone 1 (%)	Zone 2 (%)	Zone 3 (%)	Zone 1 (%)	Zone 2 (%)	Zone 3 (%)
VT_1_ (mL·kg^-1^·min^-1^)	0.74***	-0.75***	-0.49*	0.29	-0.41	0.04
VT_2_ (mL·kg^-1^·min^-1^)	0.75***	-0.71**	-0.71**	0.40	-0.40	-0.22
$\dot{V}$O_2max_ (mL·kg^-1^·min^-1^)	0.68**	-0.64**	-0.67**	0.31	-0.29	-0.19
TE (% of successful hits)	-0.33	0.32	0.27	-0.16	0.17	0.08
Competitive level (ITN)	-0.32	0.25	0.55*	-0.25	0.27	0.14

Zones defined by the VTs and the HR_max_ methods (zone 1: low intensity; zone 2: moderate intensity; zone 3: high intensity).

VT_1_, first ventilatory threshold; VT_2_, second ventilatory threshold; $\dot{V}$O_2max_, maximal oxygen uptake; TE, technical effectiveness; ITN, International Tennis Number. VTs method: Zone 1, $\dot{V}$O_2_ < VT_1_; Zone 2, VT_1_ < $\dot{V}$O_2_ < VT_2_; Zone 3, $\dot{V}$O_2_ > VT_2_. HR zones method: Zone 1 Zone 1 < 70% HR_max_; Zone 2, 70–85% HR_max_; Zone 3, > 85% HR_max_.

*p < 0.05.;

**p < 0.01.;

***p < 0.001.

## Discussion

The main finding of this study were that during a tennis set simulation, players spent on average 77% of the time in their low intensity VT zone (below VT_1_), 20% in their moderate intensity zone (between VT_1_ and VT_2_), and only 3% in the high intensity VT zone (beyond VT_2_). In addition, the time spent in the three intensity VTs zones correlated with players’ aerobic fitness parameters, meaning that players showing better aerobic fitness levels were able to play at relatively lower intensities.

### Specific endurance tennis test

On-court specific endurance tennis tests have been proposed because of their good content and ecological validity (i.e., specific sport context, tennis-specific movements), with the added value of providing useful and validated physiological parameters of aerobic fitness such as $\dot{V}$O_2max_ and the ventilatory or lactate thresholds [2]. The test used here [15,34] allows determining these parameters and provides additional information of high practical value, the TE (i.e., percentage of successful hits), which quantifies the technical effectiveness of the players along the test. This parameter has shown to explain 37% of variability in competitive performance in 38 high-level tennis players, increasing to 55% when combined with VT_2_ [15]. Moreover, VT_2_ has shown to highly correlate with $\dot{V}$O_2_ at the heart rate deflection point (r^2^ = 0.92, SE_E_ = 0.17 mL·kg^-1^·min^-1^) [34]. Thus, associating the physiological and technical parameters provides a more specific measurement of physical performance in tennis context.

Technical and physiological performance to the incremental endurance test was in the range to those obtained in a previous study conducted with tennis players of similar level and using the same test [15]. Moreover, physiological test results are also similar to values obtained in previous studies using different on-court endurance tennis tests [17,18,35].

### Simulated tennis sets

During the tennis set simulation, players were competing against opponents possessing similar technical/tactical levels. Interestingly, although there was a homogeneous sample of players, players who were not carrying the portable gas analyser won 70% of the sets. Although during the development of the on-court test, no significant effects can be attributed to wearing the portable analyser on physiological or technical parameters [15], it is possible that carrying these portable devices can have a negative effect on the players’ general performance in a playing situation (e.g., number of winning points, more unforced errors). However, because no activity profile was conducted during the present study, only speculations are possible, and the underlying limitations induced by the portable gas analyser during competitive simulation remain hypothetical.

Regarding the physiological responses during the playing simulation, average values were similar to those reported by previous studies conducted under simulated or real competitive conditions [3,4,9,10,36], with values ranging from 30 ± 4 mL·kg^**-1**^·min^**-1**^ corresponding to 52 ± 9% $\dot{V}$O_**2max**_ and 136 ± 16 b·min^**-1**^ corresponding to 70 ± 8% of HR_**max**_. Slight differences can be found in the players responses (i.e., $\dot{V}$O_2_), which may be due to differences in the evaluation protocols (i.e., specific endurance testing vs. laboratory testing), competitive level of the opponents, style of play, or the surface [1]. In this regard, differences have been observed in the activity profile and the physiological responses of the same players, but playing on different surfaces [19,37], with different tactical strategies [9], or depending of the playing situation (i.e., serving or returning) [3,10].

One of the novel aspects of the present study was to describe the relative intensity profile of a tennis set simulation, based on maximal ($\dot{V}$O_2max_ and HR_max_) and submaximal (VT_1_, VT_2_, and % HR_max_) ventilatory and HR parameters as intensity demarcation points. In most studies until now, the intensity profile in tennis play has been described as fixed percentages of $\dot{V}$O_2max_ or HR_max_ [3,4,10,12,19], although some recommend that internal load should not be based solely in relation to these two parameters [20 **–** 22]. Despite the clearly submaximal average intensity during match play (i.e., mean HR and $\dot{V}$O_2max_ values range between 60–80% HR_max_ and 50–60% $\dot{V}$O_2max_) [1,5,11], during long and intense rallies blood lactate levels can rise up to ∼9 mmol·l^-1^ [1,5,11] and HR values over 95% HR_max_ [37], clearly indicating high intensity demands requiring the activation of the anaerobic (glycolytic) metabolism.

Results showed that players spent most of the time during the set (77 ± 25%) at their low level VT zone (below VT_1_). The reason for this amount of low intensity exertion could be related to the rest periods during the game, as it is well known that the effective playing time (the real playing time (sum of all the rally durations) divided by the total match duration multiplied by 100) during a tennis match play amounts to approximately 20–30% on clay courts and to 10–15% on hard court surfaces [1]. Analysing the percentage of time in VT zone 2, players spent 20 ± 21% of the whole set time at these moderate intensities, which is similar to the previously mentioned values regarding effective playing time (15–30%) [1]. Regarding the percentage of time spent in VT zone 3 (high intensity) results showed a small amount of high-intensity exertion (3 ± 5%). Although the amount of time spent at VT zone 3 is relatively low, peak physiological values observed during play (e.g., 97% $\dot{V}$O_2max_ and 100% HR_max_) highlight the intermittent nature of tennis match play, where decisive moments (e.g., a break point) of high intensity can occur. Furthermore, in the present study just one set was analysed, and we can only speculate that a potential change in the percentage of time in the intensity zones can occur during a regular tennis match. It is possible that as the match progressed during a regular tennis match (2^nd^ and 3^rd^ set), a prolonged match imposes a significant internal load and the percentage of time in moderate and high-intensity VTs zones increase due to an increase of fatigue, not being only one set representative of the intensity elicited during a real match. In this line, it has been observed that in a four-set of elite-level tennis match, the players’ RPE, mean HR, and times in high HR zones (>85% HR_max_) all increased as the match progressed, showing increasing perceptual and physiological stress [12]. Interestingly, the authors report an upward drift in the proportion of time spent in the moderate and higher HR zone by both players as the sets progressed, suggesting the occurrence of cardiovascular drift. In this regard, the description of high-intensity periods during a female tennis tournament revealed that players spent about 13% of the total match time at exercise intensities higher than 90% HR_max._ Therefore, players must be prepared to perform high intensity exercise and to recover rapidly from it [38].

Only one study was published regarding the intensity distribution during a prolonged (4 sets) simulated tennis match in two elite professional players, based on the same HR-based zones [12]. The total duration of the match play was 197 min, and no gas analysis was performed and therefore, comparisons are difficult. However, from Fig 2 (op. cit.), it can be estimated that in the first set, the time percentages in the three HR zones were, approximately, 47/45/8% for one player, and 72/25/3% for the other. These results fit well within the range of values in our study, with the second player being very close to the average. The average HR during the first set was 137 and 128 beats·min^-1^ (no SD provided), respectively, very close to our results (138 beats·min^-1^). In this regard, we also calculated the intensity distribution in zones defined by the HR zones method in order to determine if there were differences between a VTs zones, and therefore if they were exchangeable. The VTs zones method relies on the use of sophisticated ergospirometry equipment, and the HR zones method is a simple and inexpensive method for intensity evaluation and would be more easily applicable for coaches. However the results show significant differences between both methods. Using the HR zones method the average intensity was higher, increasing the percentage of time in zone 2 and decreasing in zone 1. Differences might be related to the HR responses during intermittent exercise, as does not always reflect $\dot{V}$O_2_ variations during a playing situation (e.g., the HR to $\dot{V}$O_2_ ratio is increased during recovery periods) [39]. This is exemplified by HR showing similar values during rallying and recovery, or even slightly increased during the recovery periods between rallies [40]. In any case, the intensity zones defined using both methods were very dissimilar and, thus, not exchangeable (Fig 3). Therefore, it appears that the use of VTs zones method, which relies in two individually determined demarcation points, is more suitable for determining the relative intensity during tennis play.

### Relationship between physiological variables, competitive level and intensity zones

One of the main findings of this study was that VTs zones were correlated with the physiological parameters of tennis players. The moderate to high positive correlation between the physiological parameters (VT_1_, VT_2_ and $\dot{V}$O_2max_) and the percentage of playing time spent in VT zone 1, and the low to high inverse correlations between this variables and the percentage of time spent in VT zone 2 and 3 (Table 3), suggest that players with better aerobic fitness participate at a lower relative metabolic intensity. In contrast, no significant relationships were found between aerobic fitness and the time spent in the HR zones, which adds an argument against the use of this method to determine intensity distributions and, perhaps, also training zones. This is likely to be caused by the lack of precise physiological demarcation points when standard HR percentages are used to define the zone boundaries.

In tennis, the relationship between physiological parameters and performance has seldom been investigated and such links between isolated physical capacities and performance have not yet been well established. This is not unexpected, since successful tennis performance requires a complex interaction of physical capacities and metabolic pathways (i.e., aerobic and anaerobic) [1], thus the most sensitive physical characteristics of performance have to be identified. One study found a strong inverse relationship between $\dot{V}$O_2max_ and ATP entry ranking over time in a professional tennis player [16]. As mentioned before, while TE explained 37% of the variability in performance in a large group of high-level players [15], the present results showed no significant correlations between the TE during the test and the percentage of time spent at VTs zones. Therefore, although technical effectiveness has been identified as a good parameter to predict the competitive performance of tennis players [15,41], it seems that a better TE does not ensure an effective participation at a lower metabolic intensity of play, which emphasizes the multifaceted nature of the sport.

Another interesting finding was that a positive correlation existed between the main aerobic fitness parameters (i.e., $\dot{V}$O_2max_, VT_1_ and VT_2_) and the time spent in the low-intensity VT zone, whereas an inverse correlation was found between the former and the time spent in VTs zones 2 and 3 (moderate to high). There was also a strong inverse correlation between players’ $\dot{V}$O_2max_ and the %$\dot{V}$O_2max_ attained during play. Collectively, these findings imply that players exhibiting better aerobic fitness levels played at relatively lower intensities. This suggests that a higher level of aerobic fitness gives the player the advantage of exercising with a lower level of fatigue, which, in many instances, allows the player to sustain his technical ability for longer time. In addition, although no correlations were found between the ITN and the TE and the physiological variables, the positive correlation between the ITN and the % time spent in VT zone 3 (r = 0.55; p = 0.023), suggests that the better players (smaller ITN number), participate a small percentage of time through high physiological intensities and therefore with a lower level of fatigue. This can be due to the fact that the better players are technically and tactically more efficient at high exercise intensities. The low correlation found between ITN and physiological variables contrast with a previous study showing positive correlations between ITN and both $\dot{V}$O_2max_ and VT_2_ (r = 0.55; p = 0.001) [15]. This may be attributed to the greater homogeneity and higher level of the players in the present sample (ITN 1–3) compared to the previous study (ITN 1–4).

### Study limitations

First, as previously discussed (Methods), it is not possible to discard that carrying the portable gas measuring devices could have a negative effect on the players’ technical performance during the simulated sets. However, we can speculate but not prove that, since no influence was detected during the specific endurance tennis test in a previous study [15], the effect on the physiological variables during simulated play is likely to be relatively small. Second, a real match can be played to the best of 3 sets (a player needs to win 2 sets to win the match) or to the best of 5 sets (a player needs to win 3 sets to win the match). As discussed above, for logistic and cost constraints we monitored only 1 set during 2 separate testing sessions (i.e., with and without the portable analyzer). Therefore, it is possible that the inferior duration of the simulated match play could have an impact on the intensity of exertion compared to that elicited by a regular match, particularly due to accumulated fatigue and cardiovascular drift occurrence during longer matches [12]. Future studies should include match plays of longer duration (3–5 sets), although that would certainly influence the subjects’ tolerance to the experimental conditions.

### Practical applications

Based on the present results and according to the principle of training specificity, during specific technical-tactical tennis preparation players are to spend most of the training time at the low intensity zone (VT zone 1), and during the effective playing times they should exercise mainly within the moderate intensity zones (VT zone 2), with only some high-intensity peaks in the VT zone 3 (e.g., such as in long rallies). On the other hand, coaches should be aware that an adequate aerobic fitness of tennis players can lead to participate during the game at a lower physiological intensity and therefore at a lower level of strain and fatigue and higher technical skill. Therefore, it is advisable to periodically include training focused on improving and maintaining cardiorespiratory fitness, for which specific high-intensity interval exercises have been proposed [1,6,15,34,38].

In conclusion, this is the first study that defines the relative intensity of a singles tennis match play using three differentiated metabolic zones defined by submaximal (VT_1_, VT_2_) ventilatory parameters evaluated through a specific endurance test. The main findings of this study were that, (i) during a tennis set simulation, players spent on average 77% of the time in their low intensity zone (below VT_1_), 20% in the moderate intensity zone (between VT_1_ and VT_2_), and only 3% in the high intensity zone (beyond VT_2_), (ii) the intensity zones defined using the VTs zones method and the HR zones method were very dissimilar and, thus, not exchangeable; (iii) the time spent in the three VTs zones correlated with players’ aerobic fitness parameters, whereas the time spent in the HR zones were not, and (iv) players showing better aerobic fitness levels played at relatively lower intensities.
